# Short-term forecasts of streamflow in the UK based on a novel hybrid artificial intelligence algorithm

**DOI:** 10.1038/s41598-023-34316-3

**Published:** 2023-04-29

**Authors:** Fabio Di Nunno, Giovanni de Marinis, Francesco Granata

**Affiliations:** Department of Civil and Mechanical Engineering (DICEM), University of Cassino and Southern Lazio, Via Di Biasio, 43, 03043 Frosinone, Cassino Italy

**Keywords:** Hydrology, Civil engineering, Environmental sciences

## Abstract

In recent years, the growing impact of climate change on surface water bodies has made the analysis and forecasting of streamflow rates essential for proper planning and management of water resources. This study proposes a novel ensemble (or hybrid) model, based on the combination of a Deep Learning algorithm, the Nonlinear AutoRegressive network with eXogenous inputs, and two Machine Learning algorithms, Multilayer Perceptron and Random Forest, for the short-term streamflow forecasting, considering precipitation as the only exogenous input and a forecast horizon up to 7 days. A large regional study was performed, considering 18 watercourses throughout the United Kingdom, characterized by different catchment areas and flow regimes. In particular, the predictions obtained with the ensemble Machine Learning-Deep Learning model were compared with the ones achieved with simpler models based on an ensemble of both Machine Learning algorithms and on the only Deep Learning algorithm. The hybrid Machine Learning-Deep Learning model outperformed the simpler models, with values of R^2^ above 0.9 for several watercourses, with the greatest discrepancies for small basins, where high and non-uniform rainfall throughout the year makes the streamflow rate forecasting a challenging task. Furthermore, the hybrid Machine Learning-Deep Learning model has been shown to be less affected by reductions in performance as the forecasting horizon increases compared to the simpler models, leading to reliable predictions even for 7-day forecasts.

## Introduction

River discharge forecasting plays an essential role in flood protection and water resources planning and management. River flows are increasingly influenced by the climate changes observed in recent decades, which are leading to increasingly frequent flood and drought events^1^. In this scenario, optimal water resource management cannot disregard the prediction of river flows in the short and long term. However, while for the long term the considerable uncertainty of forecasts means that only trends can be reliably defined, for the short term it is possible to obtain even very accurate forecasts. These predictions can be conducted using different approaches, including physically based models, which consist of various mathematical equations used to describe hydrological processes^2,3^, and conceptual models, which describe the same processes based on simplified equations and empirical relationships between parameters^4^. However, the high uncertainty and complexity associated with hydrological processes and weather-climate factors affecting river basins have led researchers to increasingly use data-driven approaches, in particular Artificial Intelligence (AI) algorithms, which guarantee fast processing without the need to define complex analytical relationships between input and target variables^5^. AI algorithms have been widely applied in recent years to tackle various hydrological problems^6,7^. Among these, several Machine Learning (ML) algorithms were used for the prediction of streamflow rate^8–12^. In addition, to improve streamflow predictions, in the last few years researchers have moved towards the development of so-called hybrid or ensemble models, based on the combination of different individual ML and optimization algorithms. Li et al.^13^ compared three different ML algorithms: Back-Propagation Neural Network (BPNN), Support Vector Regression (SVR), and Adaptive Neuro Fuzzy Inference System (ANFIS), for the daily streamflow rate prediction for the Yuetan Basin, China. In particular, the authors applied the wavelet threshold de-noising method as pre-processing for time series. Then, both BPNN and SVR were combined with the Particle Swarm Optimization (PSO) algorithms. They showed how the PSO-SVR model showed a better overall performance compared to both PSO-BPNN and ANFIS models. Pham et al.^7^ proposed a hybrid model based on a ML algorithm, the Multi-Layer Perceptron (MLP), and an Intelligent Water Drop optimization algorithm (MLP-IWD) for the river flow rate forecasting of the Vu Gia Thu Bon River, Vietnam. The authors compared the predictions made with the individual MLP algorithm and the ensemble MLP-IWD, showing how hybridization led to a marked increase in performance. Saraiva et al.^14^ presented a comparative analysis of two ML models: Artificial Neural Network (ANN) and Support Vector Machine (SVM), coupled with wavelet transform and data resampling with the bootstrap method, applied for the daily streamflow rate forecasting for Sobradinho Reservoir, Brazil. The authors showed that the best combination was the BWNN, obtained combining Bootstrap (B), Wavelet (W) and Neural Network (NN), highlighting the advantages of the ensemble approach. Tyralis et al.^15^ developed a super ensemble model for one-step-ahead daily streamflow forecasting on 511 basins located in USA, based on 10 different ML algorithms. The super ensemble learning algorithm outperformed all individual ML algorithms, with, however, NN which provided the best prediction among the 10 individual algorithms. Kumar et al.^16^ compared the performance of two data-driven techniques, a Wavelet ANN (WANN) and a SVM with linear and radial basis kernel functions (SVM-LF and SVM-RF), for the daily discharge prediction of a Perennial River, India. The authors showed how SVM-RF outperformed both WANN and SVM-LF models. Kumar et al.^17^ also compared the performance of five different data-driven techniques: ANN, WANN, SVM, Wavelet SVM (WSVM) and Multiple-Linear Regression (MLR), for the forecasting of daily suspended sediment concentration in Indian Rivers, with the WSVM that outperformed the other four techniques.

Moreover, as the great potential of Deep Learning (DL) algorithms in the prediction of time series is now well known, a number of researchers have developed streamflow prediction models based on them in recent years. Fu et al.^18^ proposed a DL model based on LSTM to predict the streamflow of the Kelantan River, Malaysia. They compared the performance of the LSTM model with that of a classical neural network with back-propagation and found a higher accuracy of the LSTM model in predicting both regular flow and rapid fluctuations in the dry and rainy seasons, respectively. Le et al.^19^ presented a comparative analysis of six DL models, including: Feed-Forward Neural Network (FFNN), Convolutional Neural Network (CNN), and four Long Short-Term Memory (LSTM) -based models, applied for streamflow forecasting in the Red River basin, Vietnam. They also compared the performance of two simpler LSTM and Gated Recurrent Unit (GRU) models, with only one hidden layer, with two more complex models, the Stacked-LSTM model and the Bidirectional LSTM (Bi-LSTM) ones. The authors indicated how the LSTM models outperformed both FFNN and CNN models. However, the higher complexity of the Stacked-LSTM and Bi-LSTM models did not lead to a significant performance increase compared to the simpler LSTM models. Ahmed et al.^20^ proposed a hybrid model based on the LSTM algorithm, used in conjunction with the Boruta Feature Selection (BRF) algorithm for the optimal choice of predictors, and applied it to the prediction of streamflow forecasting in six rivers in the Murray Darling Basin, Australia. They compared the performance of the BRF-LSTM model with other ML/DL -based models: individual LSTM, GRU, Recurrent Neural Network (RNN) and SVR, with the BRF-LSTM model that outperformed all the other models. Granata et al.^21^ proposed a comparison between two different models for the daily streamflow forecasting: an ensemble model based on Random Forest (RF) and Multilayer Perceptron (MLP), hybridized using the Stacking ML technique, and a Bi-directional Long Short-Term Memory (Bi-LSTM) network, where for both the hyperparameters were optimized based on a Bayesian process. The authors showed how the ensemble model outperformed the Bi-LSTM network in predicting peaks of flow rates, with also computation times significantly shorter. Wegayehu and Muluneh^22^ also compared three DL algorithms: Stacked-LSTM, Bi-LSTM and GRU, with the classical MLP network for one-step daily streamflow forecasting for the rivers Abay and Awash, Ethiopia. They showed how both MLP and GRU algorithms outperform S-LSTM and Bi-LSTM on a nearly equal basis. A comprehensive review of the hybrid artificial intelligence and optimization modelling for streamflow forecasting was provided by Hassan Ibrahim et al.^23^.

Current literature, including a recent study by the authors mentioned above^21^, shows that reliable streamflow prediction models can be obtained using both hybrid ML and DL algorithms. Hence the idea of a possible ML-DL hybridisation with the aim of improving forecasts for both periods with ordinary flow rates and during flood events. Moreover, a further essential aspect is the forecasting horizon, which is a key element in the management of flood events. Accordingly, the performances of the developed models were assessed for forecast horizons up to 7 days. In this work, a novel prediction model was therefore developed based on the hybridization of a particular DL-RNN algorithm, the Nonlinear AutoRegressive network with eXogenous inputs (NARX), with the two algorithms RF and MLP. To the authors' knowledge, no study in the literature proposes a hybrid model based on NARX, MLP and RF for the streamflow rate forecasting. NARX networks have proven to be a valuable tool for forecasting time series of several hydrological quantities^24^. On the other hand, RF and MLP considered individually do not represent excellent solutions to the problem of forecasting hydrological time series, however, their combination can in some cases even outperform a very powerful algorithm such as LSTM networks^21^.

From this perspective, the prediction made with the hybrid NARX-MLP-RF model were compared with the ones achieved with both a model based on the single NARX algorithm and another based on the hybridization of MLP and RF. Model training, testing and subsequent comparisons were conducted as part of a large regional study, which considered the daily flow rates of 18 watercourses throughout the United Kingdom (UK). The regional scope of the comparative study represents a further innovative aspect, as UK is characterized by basins with both very different extents and characteristics of rainfall and flow regimes. Therefore, this study can provide insights into the usefulness of implementing more or less complex hybrid models depending on the features of each river.

## Materials and methods

### Case studies and dataset

**Figure 1:**
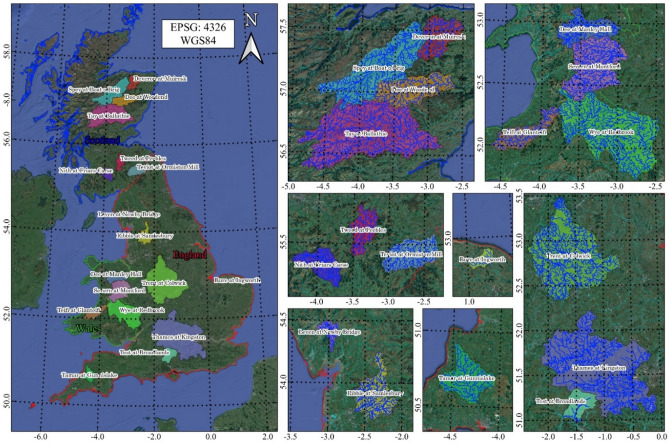
Location of the basins in UK. Maps created using the Free and Open Source QGIS^25^.

The catchment areas of the 18 rivers investigated in this study cover a significant and varied portion of the UK territory, from Scotland, where the Dee, Deveron, Spey, Tay, Nith, Teviot and Tweed rivers were analyzed, to England, where the Thames, Test, Tamar, Trent Bure, Ribble and Leven rivers were considered, and finally to Wales, where the Dee, Severn, Teifi and Wye rivers were studied (Fig. 1).

**Figure 2:**
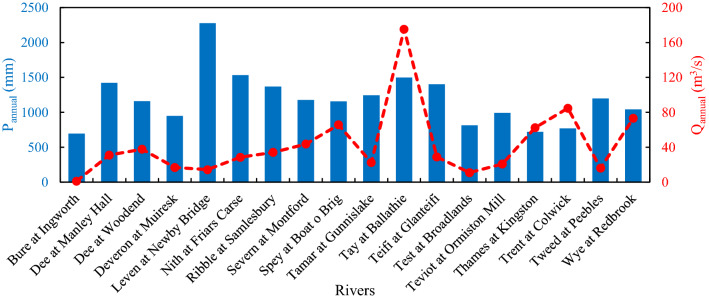
Average annual precipitation and streamflow rate.

For each measurement station, the daily cumulative precipitation and average river flow rate from January 1, 1961, to December 31, 2017, were considered. Catchment area of each basin and daily streamflow statistics were reported in Table 1. Figure 2 shows the average annual precipitation and the average annual discharge for each measuring station. The rivers investigated show considerable variability in terms of:

- Catchment area, ranging from 161 km^2^, for Bure at Ingworth (eastern England), to 9937 km^2^ for Thames at Kingston (southern England).
- Precipitation over the catchment area, ranging from an average annual precipitation (P_annual_) of 696 mm, for Bure at Ingworth, to 2277 mm for Leven at Newby Bridge (northern England). Low P_annual_ values were also observed for Thames at Kingston and Trent at Colwick, in southern and central England, equal to 723 mm and 769 mm, respectively, while high P_annual_ values were observed for the Scotland rivers of Tay at Ballathie (northern Scotland) and Nith at Friars Carse (southern Scotland), with P_annual_ of 1499 mm and 1533 mm, respectively.
- Streamflow rate: the lowest average annual discharge Q_annual_ was observed for Bure at Ingworth, equal to 1.15 m^3^/s, while the highest Q_annual_ was observed for Tay at Ballathie, equal to 175.25 m^3^/s. It should be noted that, despite Thames at Kingston has the largest catchment area of the 18 rivers, a Q_annual_ of 62.36 m^3^/s was observed, which was in line or even lower than other rivers with much smaller basins but with higher precipitations.

**Table 1:** Catchment area and streamflow rate statistics for each basin.

	Bure at Ingworth	Dee at Manley Hall	Dee at Woodend	Deveron at Muiresk	Leven at Newby Bridge	Nith at Friars Carse	Ribble at Samlesbury	Severn at Montford	Spey at Boat o Brig	Tamar at Gunnislake	Tay at Ballathie	Teifi at Glanteifi	Test at Broadlands	Teviot at Ormiston Mill	Thames at Kingston	Trent at Colwick	Tweed at Peebles	Wye at Redbrook
Area (km^2^)	161	1009	1381	962	248	798	1146	2028	2854	920	4589	898	1035	1122	9937	7472	699	4019
Q_mean_ (m^3^/s)	1.15	31.08	37.76	16.88	14.30	28.50	34.12	43.66	65.71	22.45	175.25	28.82	10.92	20.77	62.36	84.71	16.05	73.13
Q_median_ (m^3^/s)	1.01	19.24	27.23	11.43	9.81	15.89	16.90	24.20	50.20	11.70	135.70	18.19	9.58	12.15	37.90	59.30	10.26	44.70
Q_max_ (m^3^/s)	11.8	521.0	860.8	387.7	191.0	467.1	765.0	462.0	1031.0	484.0	1965.0	447.2	36.8	554.8	581.0	982.0	306.7	781.0
Q_min_ (m^3^/s)	0.38	2.75	3.54	2.06	0.11	1.15	1.88	1.71	11.28	0.58	23.07	0.73	3.78	1.41	0.01	14.70	1.85	3.43
σ_Q_ (m^3^/s)	0.60	30.86	36.38	19.14	13.70	34.59	46.42	50.01	52.15	28.86	140.47	30.36	4.87	27.02	68.31	72.59	17.16	80.97
Skew_Q_	0.72	1.15	0.87	0.85	0.98	1.09	1.11	1.17	0.89	1.12	0.84	1.05	0.82	0.96	1.07	1.05	1.01	1.05
CV_Q_	0.52	0.99	0.96	1.13	0.96	1.21	1.36	1.15	0.79	1.29	0.80	1.05	0.45	1.30	1.10	0.86	1.07	1.11
1st Q	0.79	10.55	17.03	6.74	4.25	7.36	8.60	11.60	33.54	5.09	77.33	8.47	7.39	6.15	14.90	39.70	5.93	22.44
2nd Q	1.01	19.24	27.23	11.43	9.81	15.89	16.90	24.20	50.20	11.70	135.70	18.19	9.58	12.15	37.90	59.30	10.26	44.70
3rd Q	1.33	40.21	45.05	19.87	20.20	35.94	39.80	54.50	79.41	27.70	227.50	39.22	13.25	24.13	82.00	99.40	19.59	88.80
4th Q	11.8	521.0	860.8	387.7	191.0	467.1	765.0	462.0	1031.0	484.0	1965.0	447.2	36.8	554.8	581.0	982.0	306.7	781.0

where: Area = catchment area, Q_mean_ = mean daily streamflow rate, Q_median_ = median daily streamflow rate, Q_max_ = maximum daily streamflow rate, Q_min_ = minimum daily streamflow rate, σ_Q_ = standard deviation of the daily streamflow rate, Skew_Q_ = skewness of the daily streamflow rate, CV_Q_ = coefficient of variation of the daily streamflow rate, 1st Q = first quartile of the daily streamflow rate, 2nd Q = second quartile of the daily streamflow rate, 3rd Q = third quartile of the daily streamflow rate, 4th Q = fourth quartile of the daily streamflow rate.

### Forecasting algorithms

Three artificial intelligence algorithms, NARX, MLP and RF, were considered to develop models for predicting stream flows. Subsequently, the NARX-MLP-RF hybrid model was developed in order to obtain even more accurate predictions and was compared with both the MLP-RF hybrid model and the models based on the individual algorithms. The combination of algorithms was achieved by means of the stacking technique, which allows hybrid models to be developed from multiple regression or classification models^26^. Specifically, individual models were first developed on the training dataset, then, based on the results of each model, a meta-learner was employed to develop the hybrid model. The Elastic Net algorithm^27^ was chosen as the meta-learner in the present study. Elastic Net is a combination of two widely used regularized variants of linear regression: the Least Absolute Shrinkage and Selection Operator (LASSO) and the Ridge Regression. The main difference between LASSO and Ridge is represented by the penalty (or regularization) term. LASSO uses the L_1_ regularization, with the aim of selecting the largest number of explanatory variables by introducing an absolute penalty to Ordinary Least Squares (OLS) regression. The L_1_ regularization imposes sparsity among the coefficients making the fitted model more interpretable. Ridge uses the L_2_ regularization, which also introduces a penalty in the OLS formulation, penalizing the square weights rather than the absolute ones. Moreover, the L_2_ regularization limits the size of the coefficient vector. Elastic Net represents an optimal trade-off between Ridge and LASSO, with a penalty term which is a mix of the L_1_ and L_2_ regularizations^28^, allowing to keeps the feature selection quality from the LASSO penalty as well as the effectiveness of the Ridge penalty^27^. The parameters considered for the individual algorithms are reported in Sects. "NARX model architectures", "Multilayer Perceptron (MLP)" and "Random Forest (RF)". Rainfall was used as an exogenous input for the prediction of the streamflow. Furthermore, the time series were split with a 90–10% ratio for the training and testing stages, respectively. In preliminary tests, this subdivision proved to be optimal to guarantee high performance even in the prediction of flood peaks, while still preserving a sufficiently long testing period. Therefore, the period between January 1961 and March 2012 was considered for the training stage. Then, the subsequent period between April 2012 and December 2017 was considered for the testing stage. The Bayesian Optimization (BO) procedure was used for the selection of the ML hyperparameters and the optimal number of lagged values^29^. In ML applications, the BO process aims to build a probability model of the objective function in order to select the most promising hyperparameters. For a detailed description of the BO procedure, please refer to the relevant literature^30^.

#### NARX model architectures

**Figure 3:**
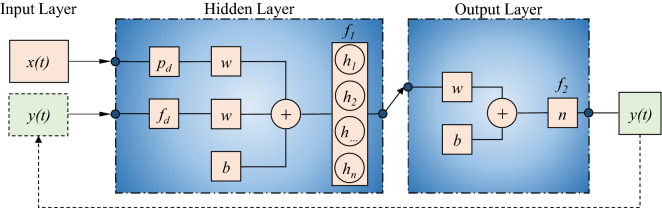
Sketch of the NARX architecture.

NARX is a particular RNN generally used for time series modeling, made up of interconnected nodes that serve as artificial neurons, receiving one or more inputs and processing them via a nonlinear activation function to produce an output. The NARX model can be formulated as:

1 $$y\left(t\right)=f\left(y\left(t-1\right),y\left(t-2\right),\dots ,y\left(t-{f}_{d}\right),x\left(t-1\right),x\left(t-2\right),\dots ,x\left(t-{p}_{d}\right)\right)$$

where *x*(*t*) and *y*(*t*) indicate the exogenous input (i.e., precipitation) and the target (i.e., streamflow rate) at time *t*, respectively, p_d_ and f_d_ that represent the precipitation and flow rates lagged values, respectively. The NARX architecture consists of three layers (Fig. 3). The first is the input layer, which receives the input parameters. The second is the hidden layer, which represents the computational stage between input and output. The third is the output layer, which provides the predicted value. Then, the estimated output was fed back as input value for the iterative computation at the next instant^31^ (dashed line in Fig. 3). For the hidden layer, a sigmoid activation function f_1_ was used, which is particularly suitable in neural networks trained through back-propagation algorithms. Moreover, the sigmoid function is derivable, making easier the neural network weights learning^32^. For the output layer, a linear activation function f_2_ with one neuron *n* was used. Weight *w* and bias *b* were optimized by means of the Bayesian Regularization (BR) back-propagation training algorithm^33^, which led to the best predictions compared with the other two training algorithms preliminarily tested, the Levenberg–Marquardt (LM) and the Scaled Conjugate Gradient (SCG). This agrees with previous literature studies that showed a slower convergence with, however, better performances for BR with respect to LM and SCG^34^.

The BO procedure led to the optimal values of both optimal number of hidden nodes (*h*_*1*_, *h*_*2*_, *h*…, *h*_*n*_, in Fig. 3) and of p_d_ and f_d_. The NARX process was stopped when one of the following conditions was met^35^: maximum number of epochs, settled equal to 1000; LM adjustment parameter, settled equal to 1 × 10^–10^; error gradient below a minimal value, settled equal to 1 × 10^–7^.

#### Multilayer Perceptron (MLP)

**Figure 4:**
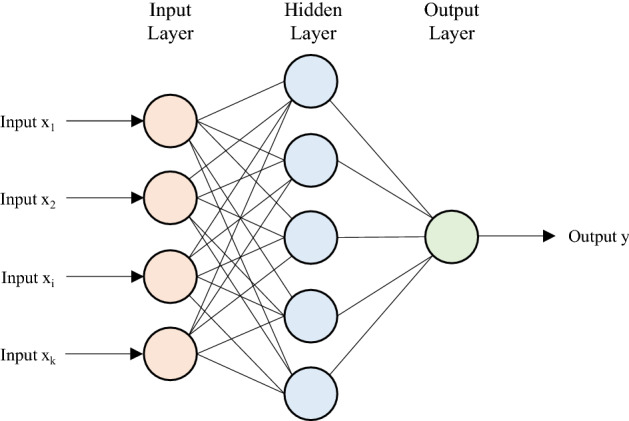
Sketch of the MLP architecture.

MLP is a particular type of feedforward ANN^36,37^ with a similar structure to NARX, with three types of layers: input, hidden, and output (Fig. 4). The input layer is made up of a set of nodes corresponding to the input variables. One or more hidden layers contain neurons that process the values included in the input layer based on a weighted linear sum followed by a non-linear activation function. Then, the output layer gets the results from the last hidden layer, providing the expected values. Backpropagation learning algorithm was used for the training of the MLP neurons. The optimal structure of the MLP network for the present study includes one hidden layer, a neuron number equal to 10, and a Sigmoid activation function. Moreover, the optimal learning and momentum rates of the backpropagation algorithm were 0.3 and 0.2, respectively.

#### Random forest (RF)

**Figure 5:**
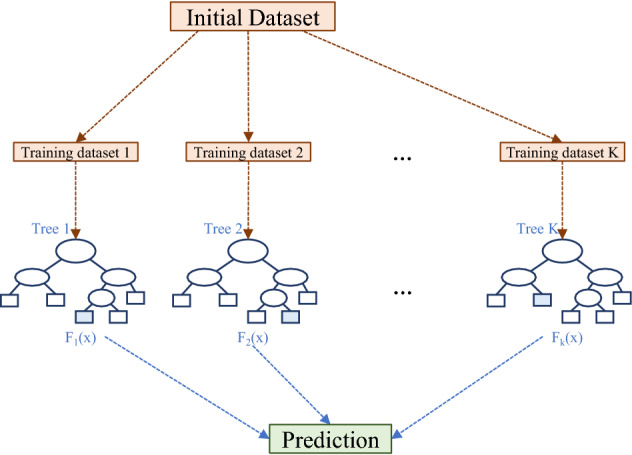
Sketch of the RF architecture.

Random Forest (Fig. 5) is an ensemble of regression tree algorithms^38^. Each tree is characterized by root and internal nodes which, respectively, include the training data and indicate the input variables conditions, and by leaves, which are the real values assigned to the target.

The development of a regression tree model consists of a recursive subdivision of the input data set into subsets, where predictions for each subset were achieved through a multivariable linear regression model. The growth of the trees is also an iterative procedure, where each subset is divided into small branches, assessing all the possible split for each field and finding, for each stage, the subdivision in two separate partitions that leads to the minimum squared deviation:

2 $$R\left({t}_{RF}\right)=\frac{1}{N(t)}\sum_{i\epsilon {t}_{RF}}\left({y}_{i}-{y}_{m}\left({t}_{RF}\right)\right)$$

where *N*(*t*) is the ${t}_{RF}$ node’s sample size, *y*_*i*_ is the target variable in the i^th^ unit, and *y*_*m*_ is the mean target variable in the node ${t}_{RF}$. *R(*${t}_{RF}$*)* provides the “impurity” at each node. The algorithm stops when the minimum impurity is reached or based on when a different stopping rule is encountered. In addition, overfitting risk is reduced through a pruning process.

It should be noted that both MLP-RF and NARX-MLP-RF models were not particularly sensitive to the number of trees, which was set equal to 100 for all rivers and models.

### Evaluation of model performance

The performance of the models was evaluated as the forecast horizon increased from 1 to 7 days ahead, based on five different evaluation metrics: the Coefficient of determination (R^2^), RMSE, the Mean Absolute Error (MAE), the Mean Absolute Percentage Error (MAPE) and the Mean Directional Accuracy (MDA). A description of the evaluation metrics is reported in Table 2.

**Table 2:** Evaluation metrics for NARX modeling.

Coefficient of determination Evaluates the goodness of fit in a regression model. It ranges between 0 (the model does not predict the outcome) to 1 (the model perfectly predicts the outcome)	${\mathrm{R}}^{2}=1-\frac{\sum_{\mathrm{i}=1}^{\mathrm{n}}{\left({\mathrm{Q}}_{P}^{\mathrm{i}}-{\mathrm{Q}}_{\mathrm{A}}^{\mathrm{i}}\right)}^{2}}{\sum_{\mathrm{i}=1}^{\mathrm{n}}{\left(\overline{{\mathrm{Q} }_{\mathrm{A}}}-{\mathrm{Q}}_{\mathrm{A}}^{\mathrm{i}}\right)}^{2}}$ (3)
Root Mean Square Error Root of total squared error between predicted and actual streamflow rate normalized by the number of samples. It ranges between 0 and + ∞ with lower values indicating more accurate models	$\mathrm{RMSE}=\sqrt{\frac{\sum_{\mathrm{i}=1}^{\mathrm{n}}{\left({\mathrm{Q}}_{P}^{\mathrm{i}}-{\mathrm{Q}}_{\mathrm{A}}^{\mathrm{i}}\right)}^{2}}{\mathrm{s}}}$ (4)
Mean Absolute Error Absolute error between the predicted and actual streamflow rate normalized by the number of samples. It ranges between 0 and + ∞ with lower values indicating more accurate models	$\mathrm{MAE}=\frac{\sum_{i=1}^{n}\left|{\mathrm{Q}}_{P}^{\mathrm{i}}-{\mathrm{Q}}_{\mathrm{A}}^{\mathrm{i}}\right|}{s}$ (5)
Mean Absolute Percentage Error Relative error between predicted and actual streamflow rate normalized by the number of samples It ranges between 0 and + ∞ with lower values indicating more accurate models	$\mathrm{MAPE}=\frac{\sum_{i=1}^{n}\left|\frac{{\mathrm{Q}}_{P}^{\mathrm{i}}-{\mathrm{Q}}_{\mathrm{A}}^{\mathrm{i}}}{{\mathrm{Q}}_{\mathrm{A}}^{\mathrm{i}}}\right|}{s}$ (6)
Mean Directional Accuracy Compares predicted and actual direction (increasing or decreasing), providing the probability that the forecasting model can detect the correct direction along the time series. It ranges between 0 and 100%, with higher values indicating more accurate models	$\mathrm{MDA}=\frac{\sum_{i=1}^{n}{1}_{sgn\left({\mathrm{Q}}_{\mathrm{A}}^{\mathrm{i}}-{\mathrm{Q}}_{\mathrm{A}}^{\mathrm{i}-1}\right) = sgn\left({\mathrm{Q}}_{\mathrm{P}}^{\mathrm{i}}-{\mathrm{Q}}_{\mathrm{A}}^{\mathrm{i}-1}\right)}}{s}$ (7)

where ${\mathrm{Q}}_{\mathrm{A}}^{\mathrm{i}}$= measured streamflow rate for the ith data and ${\mathrm{Q}}_{\mathrm{P}}^{\mathrm{i}}$ = predicted streamflow rate for the ith data, $\overline{{\mathrm{Q} }_{\mathrm{A}}}$ = mean streamflow rate, n = number of samples, sgn(·) = sign function, **1** = indicator function.

## Results

### Streamflow rate predictions on reference rivers

This section focuses primarily on flow forecasting in three reference rivers, chosen to evaluate the performance of different forecasting models in areas of the UK characterized by different rainfall regimes. The evaluation metrics for the training and testing stages, calculated for all rivers, forecasting models and temporal horizon, are shown in Tables 3, 4 and 5. In addition, Figures from 6 to 10 show the comparison between measured and predicted flow rate during the testing stage, for the different prediction models and forecast horizons.

**Table 3:**
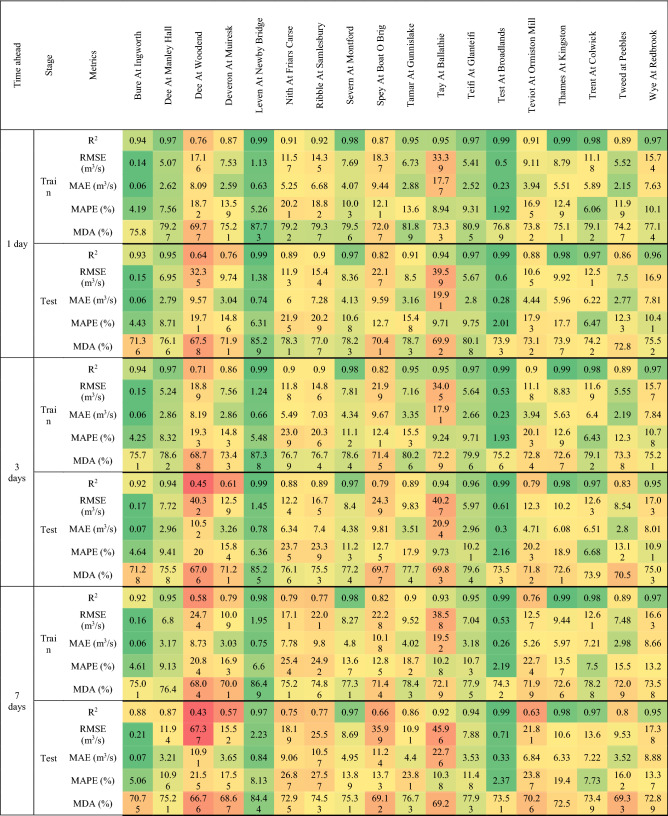
Evaluation metrics for NARX modeling.

**Table 4:**
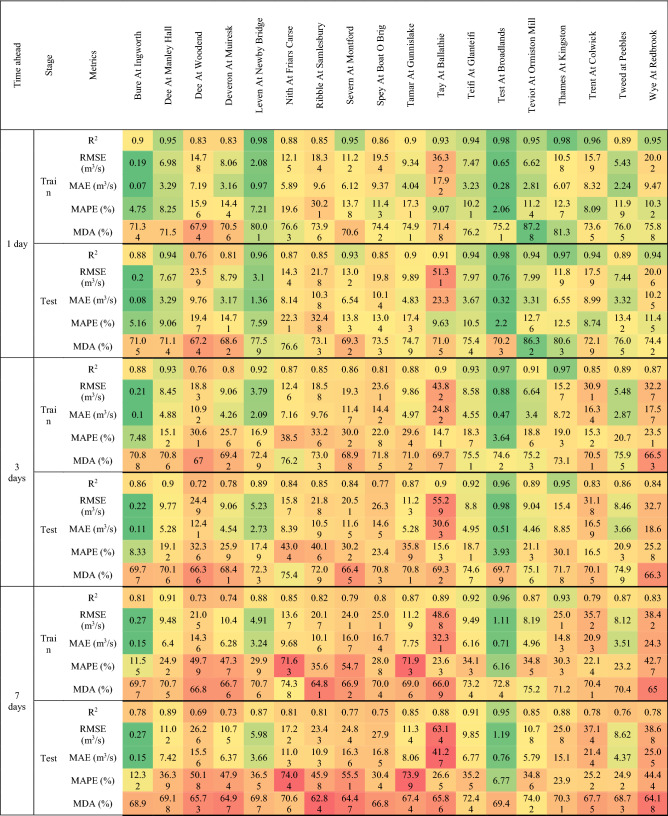
Evaluation metrics for MLP-RF modeling.

**Table 5:**
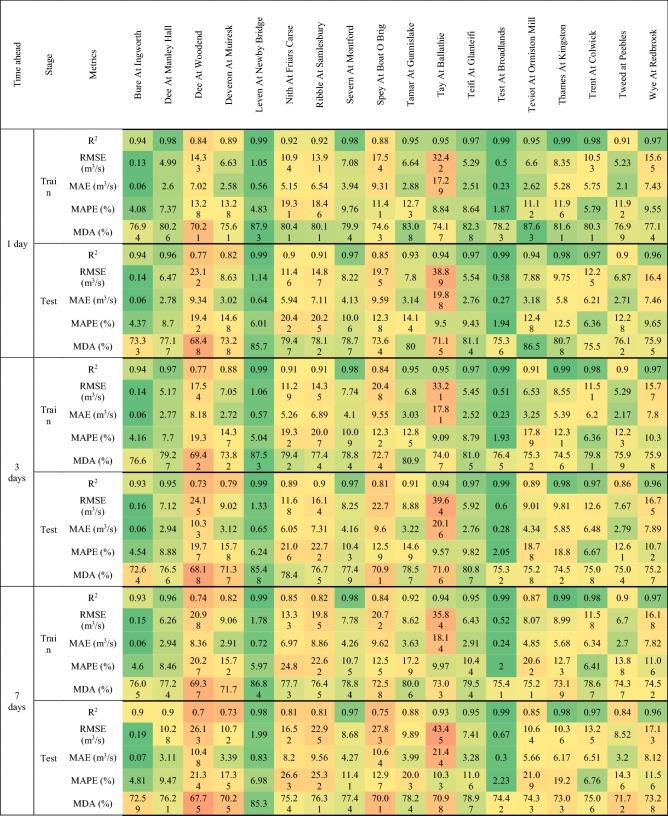
Evaluation metrics for NARX-MLP-RF modeling.

**Figure 6:**
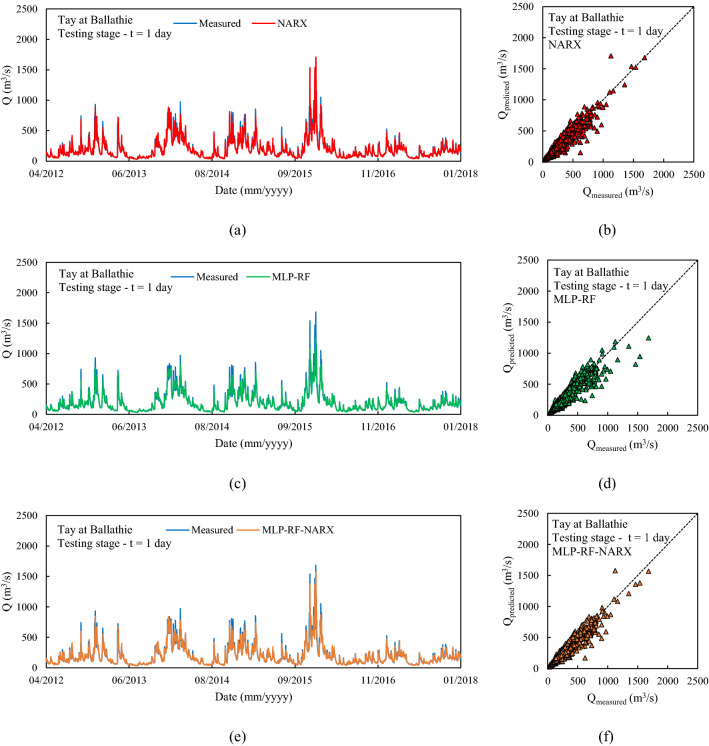
1-day ahead predictions for Tay at Ballathie: NARX (**a**, **b**); MLP-RF (**c**, **d**); MLP-RF-NARX (**e**, **f**).

**Figure 7:**
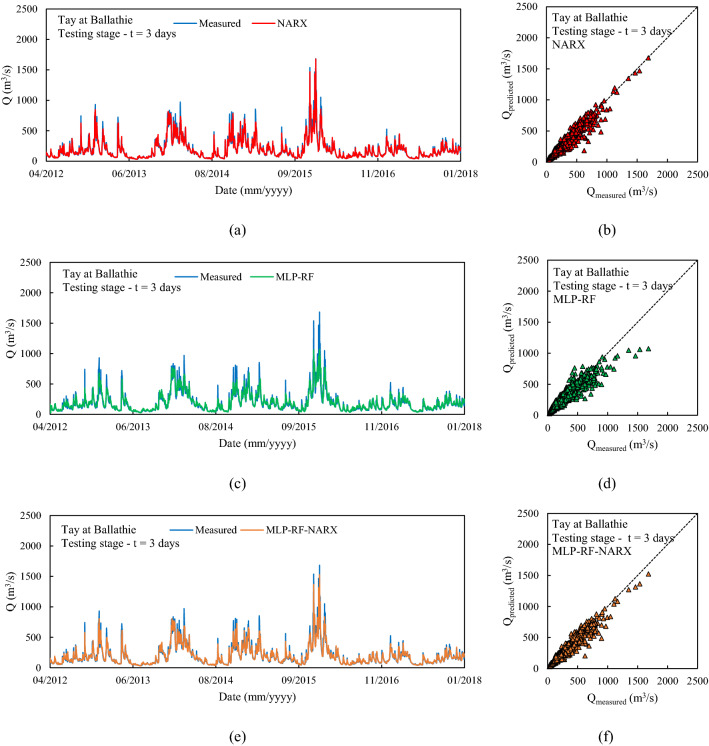
3-days ahead predictions for Tay at Ballathie: NARX (**a**, **b**); MLP-RF (**c**, **d**); MLP-RF-NARX (**e**, **f**).

**Figure 8:**
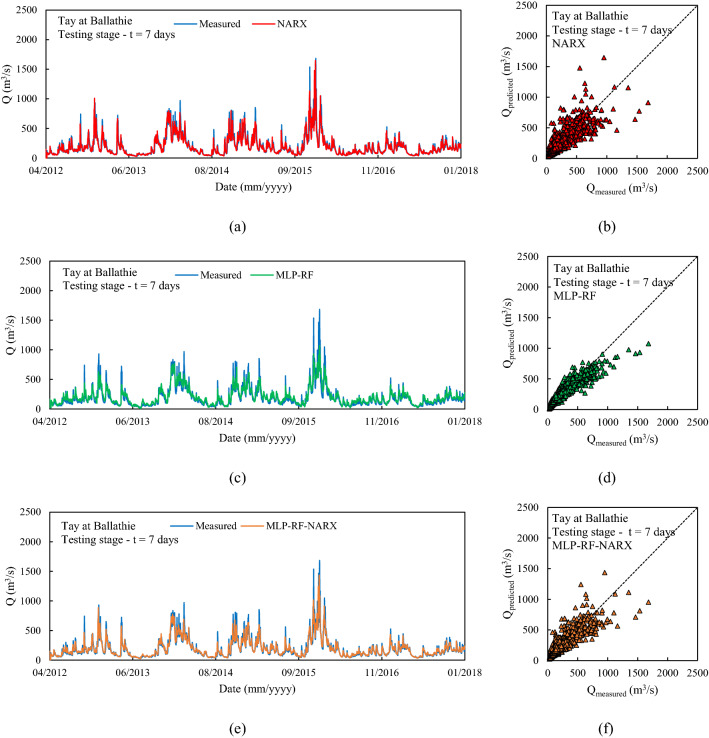
7-days ahead predictions for Tay at Ballathie: NARX (**a**, **b**); MLP-RF (**c**, **d**); MLP-RF-NARX (**e**, **f**).

The first river considered was Tay at Ballathie, Scotland, with the second highest average annual precipitation over the catchment area and the highest average annual flow rate among the 18 rivers analyzed (see Section “Case studies and dataset”). The NARX-MLP-RF hybrid model outperformed both NARX and MLP-RF models. The best performance was observed for the shortest forecast horizon t = 1 day, with the NARX model outperforming MLP-RF model for both training and testing stages. As can be seen in Fig. 6, NARX led to a more accurate prediction of the peak flow rates. However, compared to MLP-RF, NARX showed a tendency to overestimate the flow rates more frequently than MLP-RF. Therefore, the NARX-MLP-RF hybrid model, combined the advantages of both models, leading to more robust predictions compared with the two individual NARX and MLP-RF models. As the forecast horizon increases, a decrease in accuracy was observed for all models. Specifically, for t = 3 days (Fig. 7), the difference in prediction accuracy between the NARX and MLP-RF models is more marked, with the latter still showing a good ability to predict flow rate trends but with a more accentuated underestimation of the peaks, compared to t = 1 day. However, again the NARX-MLP-RF hybrid model resulted in the best forecasts, although metrics were only slightly better than the individual NARX model. The worst predictions were observed for t = 7 days (Fig. 8), with NARX showing a significant over- and underestimation of flow rates compared to shorter forecast horizons. Also, MLP-RF shows a decrease in performance with, however, a lower dispersion compared to NARX, particularly for the medium–low values of flow rate (Figur 8b and d). Consequently, the best prediction was obtained with the NARX-MLP-RF hybrid model, which showed a limited accuracy reduction from a 3-day to 7-day ahead forecast horizon.

**Figure 9:**
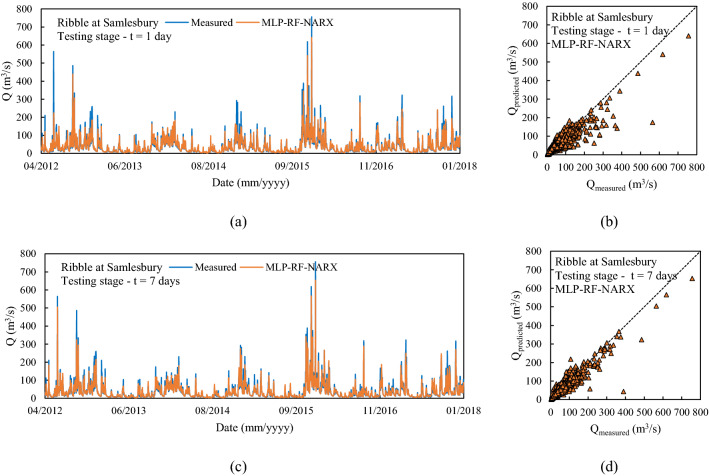
Predictions for Ribble at Samlesbury with MLP-RF-NARX model: t = 1 day (**a**, **b**); t = 7 days (**c**, **d**).

The second river analyzed in detail is the Ribble in Samlesbury, England. It showed, during the spring, a marked decreasing trend in both precipitation over the catchment area and streamflow. Figure 9 shows the comparison between measured and predicted flow rate, for forecast horizons of 1 day and 7 days, and for the NARX-MLP-RF hybrid model. Furthermore, the results for the individual models are shown in Tables 3 and 4. As for the testing stage, the best predictions were obtained for a forecast horizon of 1 day with the NARX-MLP-RF hybrid model, with R^2^ = 0.91. The NARX model (R^2^ = 0.90) resulted in slightly worse prediction than the hybrid model, while still providing more accurate forecasts than the MLP-RF model (R^2^ = 0.85). Again, as the forecast horizon increases, a reduction of the prediction accuracy was observed for the three different models. However, for t = 7 days, MLP-RF (R^2^ = 0.81) outperformed NARX (R^2^ = 0.77), which, however, still led to higher MDA values, indicating a better ability to follow the flow rate trend (MLP-RF–MDA = 62.84%, NARX–MDA = 74.53%), whereas the NARX-MLP-RF hybrid model combined the strengths of the individual models leading to better predictions (R^2^ = 0.81 and MDA = 76.31%).

**Figure 10:**
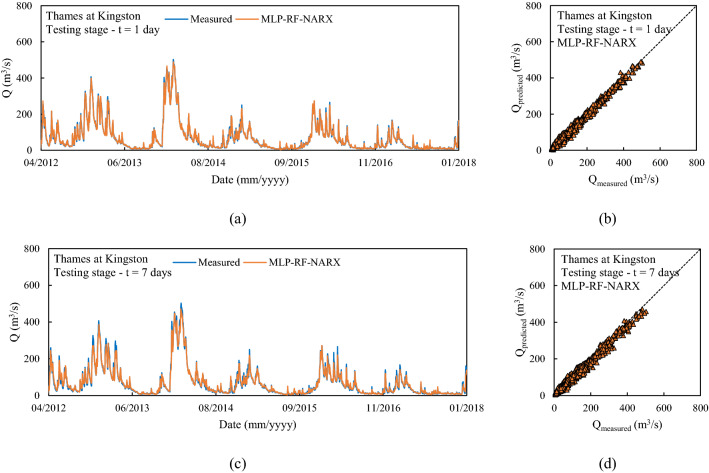
Predictions for the Thames at Kingston with MLP-RF-NARX model: t = 1 day (**a**,** b**); t = 7 days (**c**, **d**).

The third reference river was the Thames at Kingston, in the south of England, which has the largest catchment area among the 18 rivers. This case study shows overall very accurate predictions for the three different forecast models and horizons. For t = 1 day and for the testing stage, R^2^ values of up to 0.98 were calculated for MLP-RF and up to 0.99 for both NARX and the NARX-MLP-RF hybrid. The predictions became less accurate as the forecast horizon increased while maintaining higher accuracy under the same conditions, compared to the two previously investigated cases, with R^2^ values up to 0.95 for MLP-RF and 0.98 for both NARX and NARX-MLP-RF, for t = 3 days. A marked decrease was observed only for t = 7 days for MLP-RF with R^2^ = 0.88. Both NARX and NARX-MLP-RF showed an R^2^ equal to 0.98, with a limited reduction in the other metrics (Fig. 10).

Overall, the high performance of the forecast models for the Thames at Kingston can be justified by particularly gradual variations in the flow rates, which facilitate the predictions of peaks along the time series, linked to the large catchment area and lower average rainfall compared to the rest of England, and with a homogeneous distribution throughout the year. These factors make the hybridization of NARX and MLP-RF less relevant in terms of forecast improvement. Conversely, forecast models for rivers with smaller catchments and higher but less homogeneous rainfall throughout the year, as in the case of Ribble at Samlesbury, benefited more from hybridization, with better forecasts and a lower reduction in performance as the forecast horizon increases.

**Figure 11:**
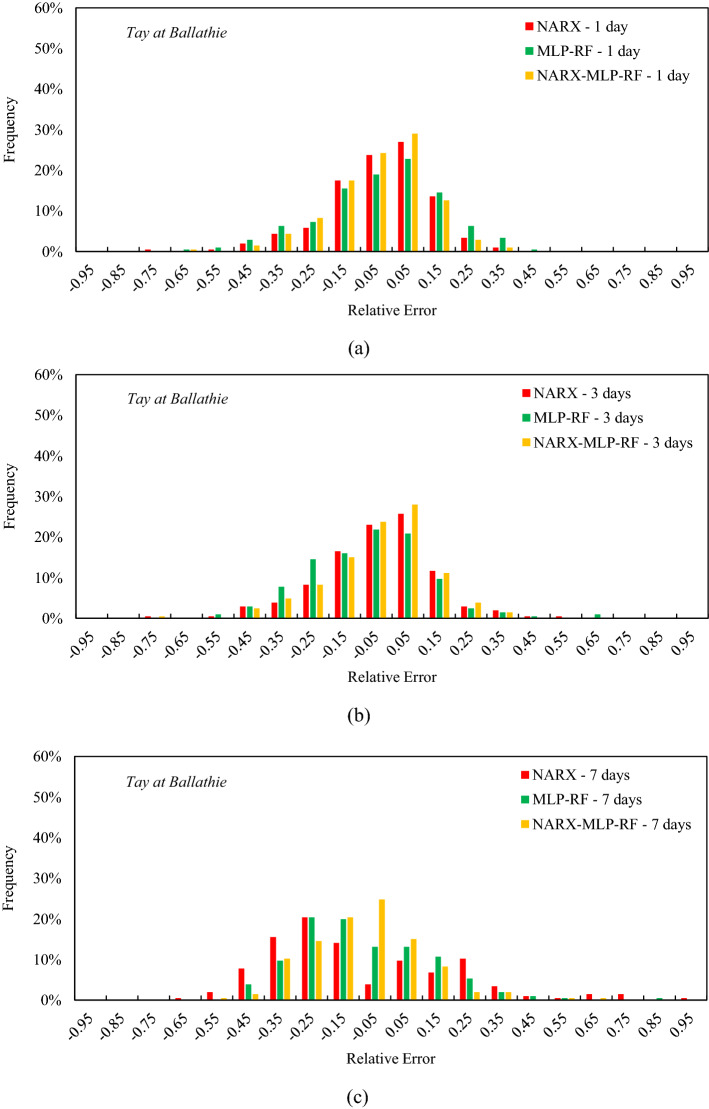
Frequency of the relative error for the tenth decile for Tay at Ballathie: t = 1 day (**a**); t = 3 days (**b**); t = 7 days (**c**).

**Figure 12:**
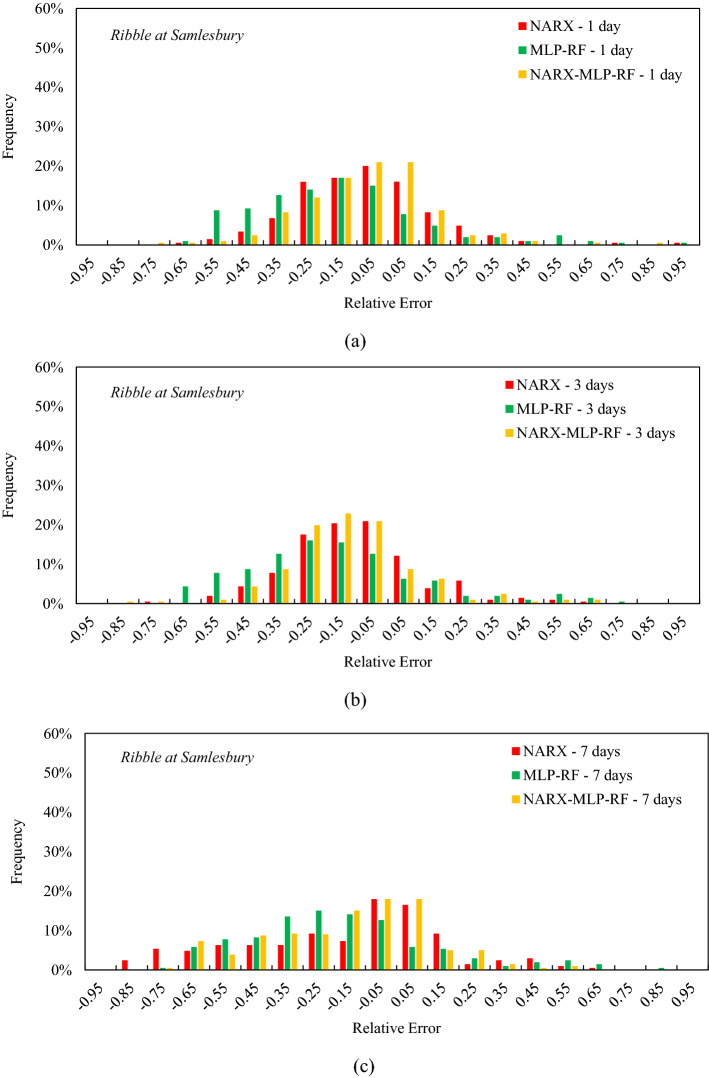
Frequency of the relative error for the tenth decile for Ribble at Samlesbury: t = 1 day (**a**); t = 3 days (**b**); t = 7 days (**c**).

**Figure 13:**
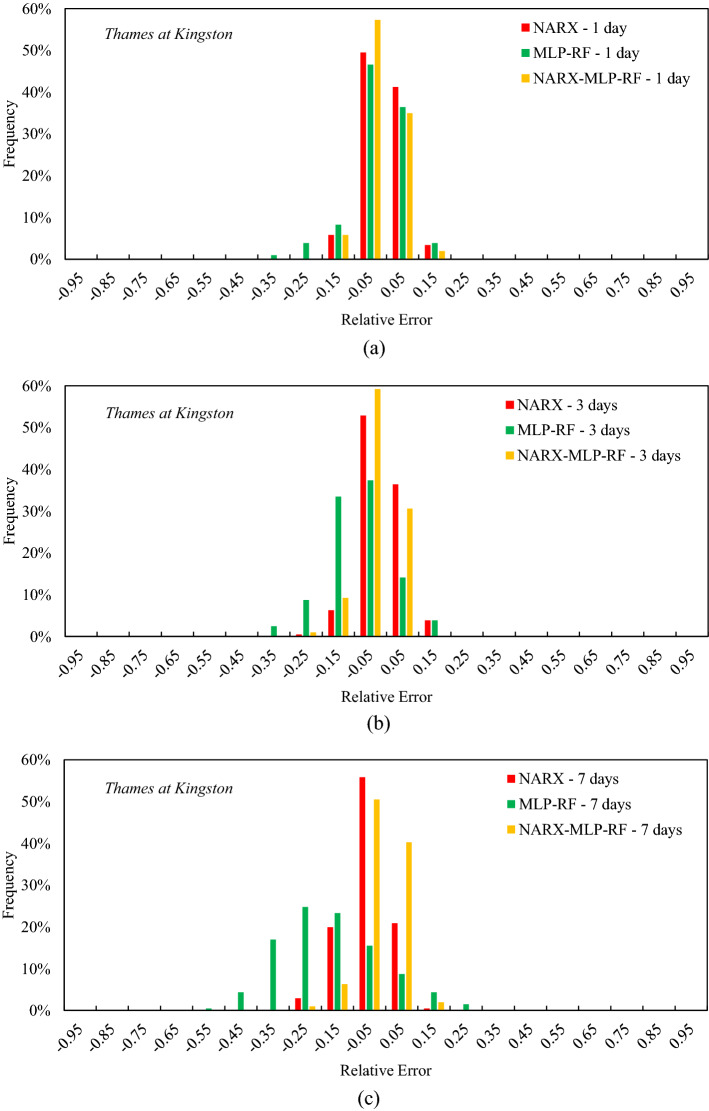
Frequency of the relative error for the tenth decile for Thames at Kingston: t = 1 day (**a**); t = 3 days (**b**); t = 7 days (**c**).

One aspect investigated with special emphasis is the highest flow rates, which can represent critical scenarios as they can lead to flooding. From this point of view, relative errors were calculated with reference to the first decile of flow rates for the three different models and for different forecast horizons. The relative errors were calculated as the difference between the predicted and measured values, divided by the measured values. Histograms with the frequency of the relative errors for the three reference rivers are shown in Figs. 11, 12 and 13, respectively. For the Tay River at Ballathie (Fig. 11) and t = 1 day, the relative errors were in the range −0.5 ÷ 0.4, with an almost symmetrical distribution for all three models. In particular, the NARX-MLP-RF ensemble model showed the highest frequency of low relative errors, equal to 24% and 29% for relative errors between −0.1 and 0 and between 0 and 0.1, respectively. MLP-RF, on the other hand, showed a lower frequency of relative errors between −0.1 and 0 and between 0 and 0.1, amounting to 19% and 23%, respectively. The NARX model showed a similar frequency distribution to the NARX-MLP-RF ensemble model with, however, slightly lower frequencies for lower relative errors. As the forecast horizon increases, the accuracy of the three models is reduced. Thus, a decrease in frequency was observed for the lower relative errors, with a subsequent increase in frequency for the higher relative errors. For t = 7 days, the NARX-MLP-RF ensemble showed the highest frequency for the relative errors between −0.1 and 0, i.e., 25%, maintaining a rather symmetrical distribution. In contrast, the NARX model showed a less symmetrical distribution with a frequency of around 20%, for relative errors between −0.3 and −0.2. Frequencies in the order of 20% were also observed for the MLP-RF model, both for relative errors between −0.3 and −0.2 (as for NARX) and between −0.2 and −0.1. This result showed a tendency for the NARX and MLP-RF models to underestimate peak flow rates.

For the Ribble at Samlesbury (Fig. 12) and t = 1 day, the relative errors were in the range -0.6–0.6. The NARX-MLP-RF ensemble showed the highest frequency of low relative errors of 21% for both relative errors between −0.1 and 0 and between 0 and 0.1, showing an almost symmetrical distribution. In contrast, MLP-RF showed a lower frequency of relative errors between −0.1 and 0 and between 0 and 0.1. The latter also showed a peak frequency of 17% for relative errors between −0.2 and −0.1, showing a more skewed distribution than the NARX-MLP-RF ensemble model. The NARX model showed lower frequencies, compared to NARX-MLP-RF, for the relative errors between −0.1 and 0 and between 0 and 0.1, amounting to 20% and 16% respectively. As the prediction horizon increased, an increase in the variance of the relative error distributions was observed, with a reduction in the frequencies corresponding to the lowest relative errors. In particular, the NARX model also showed relative errors in the range between −0.9 and −0.8, but with a very low frequency of 2%. All three models showed a higher frequency of negative relative errors, indicating that underestimates of extreme flows exceed overestimates in terms of frequency. However, the NARX-MLP-RF ensemble still showed a peak frequency of 18% for both the low relative errors between −0.1 and 0 and between 0 and 0.1.

A lower variance in relative errors was observed for the Thames first-decile flow forecasts in Kingston (Fig. 13), compared to the other two reference rivers. Specifically, for t = 1 day, the NARX-MLP-RF ensemble model showed frequencies of 57% and 35% for the lowest relative error between -0.1 and 0 and between 0 and 0.1, respectively. Furthermore, the relative errors were generally within a narrow range, between −0.2 and 0.2. MLP-RF showed a slightly worse situation, with a higher frequency of negative relative errors of 8% and 4%, between −0.2 and −0.1 and between −0.3 and −0.2, respectively. As the forecast horizon increased, the NARX-MLP-RF model still showed an almost symmetric distribution, while both NARX and MLP-RF showed an increase in the frequency of negative relative errors, resulting in a more asymmetric distribution that confirms a greater underestimation of peak flows than the NARX-MLP-RF ensemble model.

Overall, the outcomes observed for streamflow rate prediction preformed on whole time series were in agreement with what observed for the high flows. Actually, while for rivers like the Ribble, with smaller catchments and higher but less homogeneous rainfall throughout the year, relative error ranges were quite wide, for rivers with large catchments and more homogeneous rainfall like the Thames the relative error ranges were narrower, indicating a greater accuracy in the prediction of high flows. However, the hybrid NARX-MLP-RF model proves to be the best, with the NARX and MLP-RF models leading to more asymmetrical distributions even over larger basins.

### Streamflow rate predictions for the whole of UK

**Figure 14:**
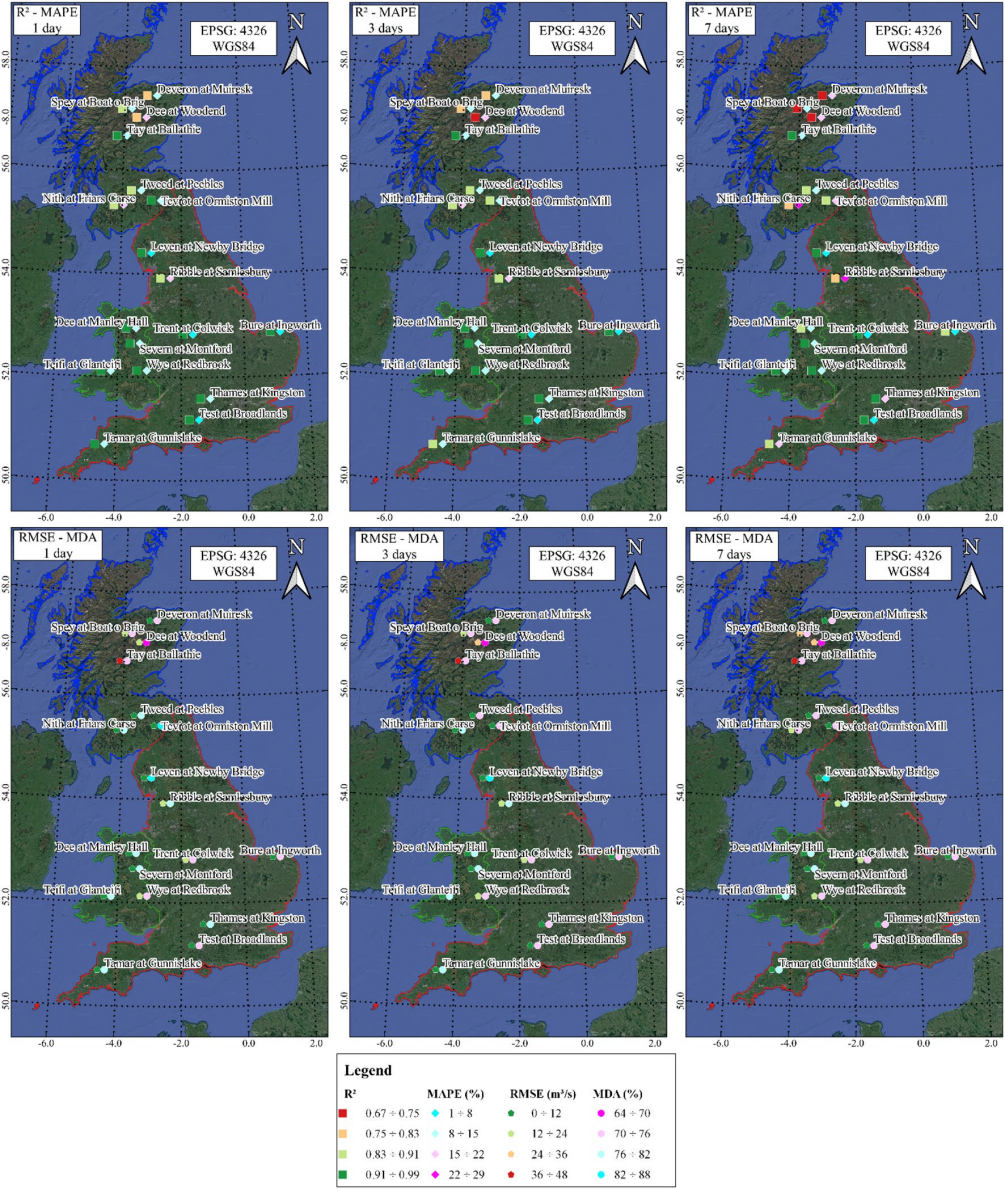
NARX-MLP-RF, testing stage: R^2^—MAPE (on the top) and RMSE—MDA (on the bottom). Maps created using the Free and Open Source QGIS^25^.

This section discusses the streamflow forecasts performed with the hybrid NARX-MLP-RF model, with reference to the testing stage, for all investigated rivers. Figure 14 provides a map with the different evaluation metrics, for R^2^–MAPE and RMSE–MDA couples, as the forecast horizon increases. Metrics are also shown in Table 5.

The R^2^ coefficient showed values ranging from 0.77 to over 0.99 for the 1-day forecasts. R^2^ decreased as the forecast horizon increased, in some cases dropping to values in the order of 0.7 for the 7-day forecast. However, there is a marked territorial difference. For rivers in the south of the UK, an R^2^ of over 0.8 was obtained, with peaks as high as 0.95, even for 7-day forecast, while for rivers in Scotland, particularly those in the north-east, lower values of 0.77 and 0.7 were obtained for the 1-day and 7-days ahead predictions, respectively. The MAPE shows a trend in agreement with the R^2^ values, with values between 1 and 26%, and increasing with the forecast horizon.

The RMSE values were consistent with the R^2^ maps, with lower values for the rivers of England and Wales, ranging from about 4 m^3^/s to 18 m^3^/s, and higher values for Scotland. The increase in RMSE as the forecast horizon increased was most pronounced for the northern UK, with RMSE up to about 40 m^3^/s for 7-days ahead predictions. However, many rivers of England and Wales were characterized by RMSE values between 4 m^3^/s and 18 m^3^/s even for 7-days ahead predictions. In addition, MDA values between 64 and 88% were calculated, showing a good ability of the forecasting model to follow the right direction along the streamflow time series. A slight reduction was observed as the forecast horizon increases, with, however values between 64 and 70% observed only for rivers in central and north-east Scotland, where the lowest R^2^ values were also obtained.

Overall, the hybrid NARX-MLP-RF model resulted in good predictions for all rivers and forecast horizon. However, the performance of the forecast model is highest for rivers with large basins and a homogeneous distribution of rainfall throughout the year, as observed for several English rivers, while it is lowest for rivers with smaller basins, characterized by less homogeneous rainfall, where peak prediction is more challenging due to the sudden variation in stream flow.

**Figure 15:**
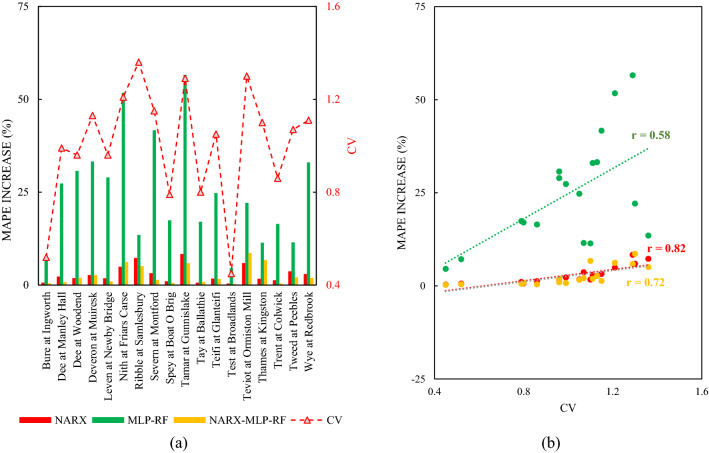
MAPE percentage increase as the forecast horizon increases: histogram for the 18 rivers (**a**); CV vs MAPE percentage increase (**b**).

In order to provide an overview of how model performance changes with the forecast horizon, the percentage increase in MAPE, from a 1-day to a 7-day forecast horizon was analysed and reported in Fig. 15.

In particular, the ensemble NARX-MLP-RF model showed the lower MAPE variations for most stations, followed by the NARX model. Both showed MAPE variations of less than 10%. In contrast, MLP-RF showed more marked MAPE variations, with a maximum value of 56% for Tamar at Gunnislake. However, for some stations, MLP-RF also showed MAPE variation of less than 10%. For example, for Test at Broadlands, the MAPE variation was 4.57%. However, for the same station, NARX and NARX-MLP-RF showed lower MAPE variations of 3.60% and 2.90% respectively. It was noted that there is an appreciable correlation between the increase in MAPE just considered and the CV of the flow time series (Fig. 15). The correlation is high for the NARX model (r = 0.82) and rather high for the NARX-MLP-RF ensemble model (r = 0.72), whereas it is significantly lower for the MLP-RF hybrid model (r = 0.58).

This result demonstrates that while the decrease in accuracy of the forecast models, as the forecast horizon increases, is proportional to the variability of the streamflow during the time series, and this decrease is much less pronounced in the NARX model than in the hybrid RF-MLP one. However, this aspect needs further investigation and specific studies.

## Discussion

The extensive study carried out on the streamflow of a large number of rivers in the United Kingdom allows the following to be highlighted:

- The NARX-MLP-RF hybrid model outperformed both the NARX and MLP-RF models for all the investigated rivers and for all forecast horizons. All models resulted in very accurate predictions in the south of UK, while lower performance was observed in the north of UK.
- A reduction in performance was observed as the forecasting horizon increased, but this affected the NARX and MLP-RF models more than the hybrid NARX-MLP-RF model.
- The hybridization of NARX and MLP-RF had a greater impact in improving the predictions obtained for small basins with high and uneven precipitation throughout the year, which make peak forecasting more challenging. Conversely, individual NARX and MLP-RF models led in most cases to satisfactory results, without the need for hybridization, for large basins with a more gradual variation in flow rates.

Regarding the application of hybrid ML models for streamflow forecasting, Li et al.^13^ developed hybrid models for the Yuetan Basin, China, achieving the best results with a PSO-SVR model, which showed a Nash–Sutcliffe Efficiency (which has a mathematical expression almost identical to R^2^) of 0.82, lower than the R^2^ values obtained for several rivers investigated in this study. The super ensemble model proposed by Tyralis et al.^15^ resulted in large differences, in terms of prediction accuracy, among the large number of investigated rivers in USA, with R^2^ values mostly between 0.60 and 0.65. Lee and Ahn^39^ developed a stacking model based on four ML algorithms: SVM, Gradient Boosting Machine (GBM), Cubist, and Bayesian Regularized Neural Networks (BRNN), for the streamflow rate prediction in South Korea. The authors calculated values of NSE up to 0.48, also showing a performance reduction as the forecast horizon increased, as observed in the present study. Kilinc and Yurtsever^40^ also developed a hybrid DL model Based on Grey Wolf algorithm (GWO) and GRU for the daily streamflow forecasting in two stations located in the Seyhan basin, Turkey. The authors showed the advantages of the hybridization based on DL algorithms, achieving accurate predictions with R^2^ values up to 0.98. The results obtained by Kilinc and Yurtsever^40^ are in line with the prediction obtained for several rivers investigated in the present study for t = 1 day. However, they did not perform an analysis with increasing time horizon, as made in the present study. Granata et al.^21^, who proposed a comparison between Bi-LSTM and a stacked MLP-RF model, obtained very accurate predictions, also for the UK Trent River investigated in this study. However, they also showed a reduction in prediction accuracy as the forecast horizon increased, already for the 3-days forecast.

A comparison was also made with literature studies investigating the impact of climatic factors and catchment characteristics on the accuracy of river discharge forecasts. Xu et al.^41^ investigated the spatial and temporal scale effects on the predictive performance of the monthly streamflow prediction, based on a hybrid DL model based on the CNN and GRU algorithms applied to many watersheds around globe. The authors showed how the hybrid DL model performs better on large drainage areas, in agreement with the present study. Moreover, the predictive performance tends to get better also with the extension of a training period for the model, confirming how long time series can lead to more accurate predictions. Harrigan et al.^42^ evaluating the Ensemble Streamflow Prediction (ESP) method for 314 catchments in the UK, exploring the relationship between basins characteristics and ESP skill. The ESP method allows factors such as precipitation, potential evapotranspiration, temperature, soil moisture, groundwater and snow for each basin to be included in the modelling. The authors showed how the performance of the ESP model decayed exponentially with increasing forecast horizon, but large catchments decayed at a slower rate. In addition, better performances were observed in the south and east of the UK, where large and slower responding catchments are mainly located. Conversely, lower performances were observed for the highly responsive catchments in the north and west. These outcomes are in agreement with the present study. We showed that for large basins, such as for the Thames River in southern England, the models tested led to accurate predictions for both ordinary and high flows, whereas for smaller basins, such as for the Ribble River in Northern England, forecasts were less accurate and decayed in accuracy at a higher rate compared to larger basins as the forecast horizon increased, particularly for the NARX and MLP-RF models.

Overall, although the methodology has been tested on a significant number of rivers, UK weather and climate conditions have different features in comparison with warmer climates. In the future it will be interesting to test the methodology in semi-arid and Mediterranean areas, where the seasonal pattern of rainfall is more pronounced compared to UK. From this perspective, different ML or deep-learning algorithms could be included, together with further exogenous inputs, in the forecast procedure in order to improve the reliability of the streamflow rate forecasting. This could lead to overcoming the current limitations related to climate and streamflow regimes on the one hand and the forecasting horizon on the other, moving from the current short-term to the medium-term scenario.

## Conclusion

A novel streamflow prediction model, for forecast horizons of up to seven days, was developed in this research and applied to a regional study that considered 18 rivers throughout the UK. The proposed model was obtained by stacking the NARX, RF, and MLP algorithms and used a BO procedure for tuning the hyperparameters.

Daily precipitations were considered as the only exogenous input variable. The NARX-MLP-RF ensemble model showed very good forecasting capabilities and outperformed both NARX and MLP-RF models, for all rivers and forecast horizons. NARX-MLP-RF showed a lower reduction of accuracy as the forecasting horizon increased, for both regular and extreme streamflow, compared to the NARX and MLP-RF models. In this regard, for both the NARX-MLP-RF and NARX models, a significant correlation was found between the increase in MAPE corresponding to the increase in the forecast horizon from 1 to 7 days and the CV of the flow time series.

In addition, NARX-MLP-RF has proven to be particularly suitable for providing accurate forecasts for rivers with small catchment areas with highly variable rainfall and streamflow rate distributions over time., for which the forecasting of the often-abrupt peaks is a challenging task. In particular, more accurate forecast values were generally obtained for rivers in Wales and southern England.

Overall, the accurate predictions made with the NARX-MLP-RF model make it a powerful tool for managing the risks associated with possible extreme flows involving frequent floods, and also for short-term water management decision-making.

## Data Availability

Data from the National River Flow Archive, which is the primary archive of daily and peak river flows for the United Kingdom, were used in the creation of this manuscript. Data are available at the following website: https://nrfa.ceh.ac.uk. The elaborations were carried out mostly with the following software: MATLAB (https://mathworks.com), Microsoft Excel (https://www.microsoft.com/en-ww/microsoft-365/excel), and QGIS (https://qgis.org/en/site).

## Abbreviations

1: Indicator function
1st Q: First quartile of the daily streamflow rate
2nd Q: Second quartile of the daily streamflow rate
3rd Q: Third quartile of the daily streamflow rate
4th Q: Fourth quartile of the daily streamflow rate
ANFIS: Adaptive Neuro Fuzzy Inference System
ANN: Artificial Neural Network
AI: Artificial intelligence
*b*: Bias in NARX model
BO: Bayesian optimization
BR: Bayesian regularization
BI-LSTM: Bidirectional Long Short-Term Memory
BRF: Boruta Feature Selection
BPNN: Back-Propagation Neural Network
BRNN: Bayesian Regularization Neural Network
BWNN: Bootstrap Wavelet Neural Network
CNN: Convolutional Neural Network
CV_Q_: Coefficient of variation of the daily streamflow rate
DL: Deep Learning
ESP: Ensemble Streamflow Prediction
f_1_: Sigmoid activation function
FFNN: Feed-Forward Neural Network
GRU: Gated Recurrent Unit
GWO: Grey Wolf Optimization
*h*: Hidden nodes in NARX model
IWD: Intelligent Water Drop
LASSO: Least Absolute Shrinkage and Selection Operator
LM: Levenberg–Marquardt
LSTM: Long Short-Term Memory
ML: Machine Learning
MLR: Multiple-Linear Regression
n: Number of neurons in NARX model
NARX: Nonlinear AutoRegressive network with eXogenous inputs
OLS: Ordinary Least Squares
PSO: Particle Swarm Optimization
$\overline{{\mathrm{Q} }_{\mathrm{A}}}$: Mean streamflow rate
${\mathrm{Q}}_{\mathrm{A}}^{\mathrm{i}}$: Measured streamflow rate for the ith data
${\mathrm{Q}}_{\mathrm{P}}^{\mathrm{i}}$: Predicted streamflow rate for the ith data
Q_max_: Maximum daily streamflow rate
Q_mean_: Mean daily streamflow rate
Q_median_: Median daily streamflow rate
Q_min_: Minimum daily streamflow rate
*R(t*_*RF*_*)*: Impurity at each node in RF model
RF: Random Forest
SCG: Scaled Conjugate Gradient
sgn(·): Sign Function
Skew_Q_: Skewness of the daily streamflow rate
SVM: Support Vector Machine
SVM-LF: Support Vector Machine with Linear kernel function
SVM-RF: Support Vector Machine with Radial basis kernel function
SVR: Support Vector Regression
s: Number of samples
t: Forecast horizon
*t*_*RF*_: Node in the RF model
UK: United Kingdom
*w*: Weight in NARX model
WANN: Wavelet Artificial Neural Network
WSVM: Wavelet Support Vector Machine
*x(t)*: Value of the exogenous input at time *t* in the NARX model
*y*_*i*_: Target variable in the ith unit in the RF model
*y*_*m*_: Mean target variable in the node *t*_*RF*_
*y(t)*: Target at time *t* in the NARX model
σ_Q_: Standard deviation of the daily streamflow rate
